# 

**Published:** 1860-11

**Authors:** 


NORTH AMERICAN

### MEDICO-CHIRURGICAL REVIEW.

Vol. IV.] NOVEMBER, 1860. [No. 6.

### CONTENTS.

### ANALYTICAL AND CRITICAL REVIEWS.

ART. PAGE

I.—Bright's Disease of the Kidneys. By FRIED. T. FRERICHS, M.D......... 961
II.—The Nature and Treatment of Gout. By A. BARING GARROD, M.D..... 988
III.—On Obscure Diseases of the Brain, and Disorders of the Mind. By FORBES WINSLOW, M.D........ 1003
IV.—Notes on Nursing. By FLORENCE NIGHTINGALE........ 1015
V.—Caloric: Its Agencies in the Phenomena of Nature. By SAMUEL L. METCALFE, M.D........ 1019
VI.—Diseases, etc. of the Rectum and Anus. By T. J. ASHTON, Surgeon... 1021
VII.—The Principles, etc. of Modern Surgery. By R. DRUITT, Licentiate... 1022
VIII.—Caffein an Antidote to Opium. By H. F. CAMPBELL, M.D............... 1023
IX.—The Anatomy, etc. of the Placenta. By JOHN O'REILLY, M.D.......... 1024
X.—How to Work with the Microscope. By LIONEL BEALE, M.D............ 1024
XI.—Skin Diseases and their Remedies. By ROBERT J. JORDAN, M.D........ 1025
XII.—An Epitome of Braithwaite's Retrospect. By WALTER S. WELLS, M.D. 1025
XIII.—Quarterly Summary of Transactions of the College of Physicians of Philadelphia........ 1026
XIV.—Transactions of the New Jersey State Medical Society for 1859......... 1027

### ORIGINAL COMMUNICATIONS.

I.—Origin of Ovariotomy; with an Account of the Life and Services of the Late Dr. Ephraim McDowell. By the SENIOR EDITOR................ 1028
II.—Cases from the Surgical Clinic of Jefferson Medical College.—Service of Professor Gross. By EMIL FISCHER, M.D........ 1053
III.—Foreign Body in the Right Bronchus; Tracheotomy; Removal and Recovery. By JOHN M. ADLER, M.D........ 1062
IV.—A Case of Procidentia Uteri. By GUSTAVUS G. ROY, M.D............... 1065
V.—Grave Sequelæ of a Renal Calculus. By W. H. TRIPLETT, M.D........ 1067

### BI-MONTHLY ABSTRACTS.

SURGERY. BY S. W. GROSS, M.D.

On Iridectomy in Glaucoma.—Inguinal and Popliteal Aneurisms cured by Digital Compression.—Dislocation of the Clavicle treated by Wiring.—Excision of the Knee-Joint.—Fracture of both Femurs by Muscular Spasm.—Excision of the Elbow-Joint and Entire Ulna.—Traumatic Inflammation of the Sinuses of the Dura Mater.—Dislocation of the Crystalline Lens into the Anterior Chamber of the Eye.—Cases of Vesical Calculus in the Norfolk and Norwich Hospital.—On Iritis as it occurs in Syphilitic Infants........ 1069

PATHOLOGY AND PRACTICE OF MEDICINE. BY RICHARD J. DUNGLISON, M.D.

Connection between Chorea and Acute Rheumatism.—Difficulties in the Diagnosis, etc. of Mental Diseases.—Pleurisy, Pericarditis, and Disease of Liver supervening in Caries of the Spine.—Case of Double Transverse Hemiplegia.—Apoplectic Affections of the Retina.—Hemorrhagic Diathesis in Phthisis Pulmomalis and other Acute or Chronic Affections.—Conditions attending every attack of Acute Rheumatism.—Statistical Contribution to the Diagnosis of Cancer of the Stomach........ 1076

OBSTETRICS, AND DISEASES OF WOMEN AND CHILDREN. BY WM. B. ATKINSON, M.D.

A New Method of Version in Abnormal Labor.—Delivery of the Placenta in Abortions in Early Pregnancy.—Case of Rupture of the Uterus.—Retroflexion of the Unimpregnated Uterus.—Phlegmasia Dolens.—Influence of Lead Poisoning on the Product of Conception. Fatal Effects from Enlargement of Thymus Gland in Children.—Treatment of Hooping-Cough by Sulphate of Zinc and Extract of Belladonna.—Convulsions in Children treated with Valerian........ 1085

PHYSIOLOGY. BY S. WEIR MITCHELL, M.D.

Coloration of the Bones of the Fœtus by the Action of Madder in the Food of the Mother.—Physiological Papers read before the British Association for the Advancement of Science........ 1093

MATERIA MEDICA AND THERAPEUTICS; MEDICAL CHEMISTRY; TOXICOLOGY AND LEGAL MEDICINE. BY EMIL FISCHER, M.D.

*Materia Medica and Therapeutics:* Stearate of Iron in the Treatment of Soft Chancres.—Hydrochloric Acid in Chronic Dyspepsia.—Hydrocotyle Asiatica in Chronic Eczema.—Piperin in Intermittent Fever.—Arsenic in Apoplectic Congestion.—Asarum Europæum a Remedy for the Effects of Drinking.—Saccharate of Colchicum in the Treatment of Gout and Articular Rheumatism.—Chloride of Iron in Cutaneous Diseases.—Ether as a Remedy for Deafness.—History and Pretensions of Cod-Liver Oil.—A New Alkaloid in Coca.—Action of Different Medicines on the Mental Faculties........ 1097

*Medical Chemistry:* On the Amount of Water contained in the White and in the Gray Substance of the Brain.—On Uroglaucine........ 1107

*Toxicology and Legal Medicine:* Means of Distinguishing Blood-Stains on a Rusty Instrument.—Experimental Researches on Death by Drowning.—Influence of External Pressure upon the Formations of Suggillations.—Researches on the Dangers presented by Schweinfurt Green, etc.—Detection and Estimation of Phosphorus and Phosphorous Acid.—Poisoning by a Dress containing Arsenic........ 1108

PATHOLOGICAL ANATOMY. BY JOHN H. PACKARD, M.D.

Embolie of the Middle Cerebral Artery.—Pneumothorax; Death from Dissecting Aneurism of the Aorta.—Transposition of the Arteries.—Cancerous Degeneration of Lung.—Intestinal Concretions formed of Oat-hairs.—Diseases of the Uterus........ 1111

### BI-MONTHLY PERISCOPE.

THERAPEUTICS.—Disinfectants........ 1118

EDITORS' TABLE.—Ancient Medicine.—Medical Missionary Society's Hospital at Canton.—The Case of Sir Renjamin Brodie.—Honors to Medical Men.—New Medical Journals.—Year-Book of American Contributions to Medical Science and Literature.—Dictionary for the Blind.—Changes and Appointments in Medical Schools.—Boylston Medical Prize Questions.—Fiske Fund Prize Essays.—Clinical Instruction at the Philadelphia Hospital.—Museum of the Philadelphia Hospital.—Pennsylvania Institution for the Instruction of the Blind.—Appointments to Insane Hospitals.—New Sydenham Society's Publications........ 1125

NECROLOGICAL NOTICES.—Dr. John Bellinger.—Dr. Samuel Denton.—Dr. Bernard M. Byrne.—Mr. John Crichton.—Dr. Leroy D'Etoilles........ 1134

BI-MONTHLY BIBLIOGRAPHICAL RECORD........ 1135

